# The J Domain of Sacsin Disrupts Intermediate Filament Assembly

**DOI:** 10.3390/ijms232415742

**Published:** 2022-12-12

**Authors:** Afrooz Dabbaghizadeh, Alexandre Paré, Zacharie Cheng-Boivin, Robin Dagher, Sandra Minotti, Marie-Josée Dicaire, Bernard Brais, Jason C. Young, Heather D. Durham, Benoit J. Gentil

**Affiliations:** 1 Department of Neurology and Neurosurgery, Montreal Neurological Institute, McGill University, Montreal, QC H3A 2B4, Canada; 2 Department of Kinesiology and Physical Education, McGill University, Room 210, 475 Pine Avenue West, Montreal, QC H2W 1S4, Canada; 3 Laboratory of Neurogenetics of Motion, Montreal Neurological Institute, McGill University, Montreal, QC H3A 2B4, Canada; 4 Department of Biochemistry, McGill University, Montreal, QC H3G 1Y6, Canada

**Keywords:** ataxia, motor neuron, neurofilament, intermediate filaments, chaperone, J domain, vimentin

## Abstract

Autosomal Recessive Spastic Ataxia of the Charlevoix Saguenay (ARSACS) is caused by mutation in the SACS gene resulting in loss of function of the protein sacsin. A key feature is the formation of abnormal bundles of neurofilaments (NF) in neurons and vimentin intermediate filaments (IF) in cultured fibroblasts, suggesting a role of sacsin in IF homeostasis. Sacsin contains a J domain (SacsJ) homologous to Hsp40, that can interact with Hsp70 chaperones. The SacsJ domain resolved NF bundles in cultured *Sacs*^−/−^ neurons. Having studied the mechanism using NF assembled in vitro from purified NF proteins, we report that the SacsJ domain interacts with NF proteins to disassemble NFL filaments, and to inhibit their initial assembly. A cell-penetrating peptide derived from this domain, SacsJ-myc-TAT was efficient in disassembling NF bundles in cultured *Sacs*^−/−^ motor neurons, restoring the NF network; however, there was some loss of vimentin IF and NF in cultured *Sacs*^+/+^ fibroblasts and motor neurons, respectively. These results suggest that sacsin through its SacsJ domain is a key regulator of NF and vimentin IF networks in cells.

## 1. Introduction

Autosomal Recessive Spastic Ataxia of the Charlevoix Saguenay (ARSACS), the second most common recessive ataxia worldwide, is a childhood-onset neurodegenerative disease caused by over 200 different mutations in the SACS gene. SACS encodes the giant 520 kDa, multi-domain protein sacsin [1,2]. Although ubiquitously expressed, sacsin is a cytoplasmic protein enriched in neurons [3]. ARSACS patients exhibit a clinical triad of ataxic gait, pyramidal spasticity, and peripheral neuropathy [4,5,6,7,8,9]. Sacsin knockout (*Sacs*^−/−^) mice exhibit a similar phenotype, supporting a loss of function mechanism [10]. 

The domain structure of sacsin, characterized based on sequence homology using in silico analysis [11], points to functions in protein chaperoning and quality control. Sacsin contains, starting at the N-terminus, an ubiquitin-like domain (UBL), which can bind to the proteasome 20S α subunit [12]; three large sacsin repeat regions (SRR), termed SIRPT1, SIRPT2, and SIRPT3, which share homology with the chaperone, Hsp90; an XPC-binding domain; and then at the C-terminus, a J domain (SacsJ) homologous to Hsp40, immediately followed by a single higher eukaryote and prokaryote nucleotide-binding domain (HEPN), believed to promote sacsin dimerization [11,13,14,15]. 

Evidence, including homology to heat shock proteins, further points to chaperone and quality control roles of various sacsin domains. The XPC-binding domain of another protein, HR23A, binds to the ubiquitin ligase Ube3A, suggesting that sacsin might be ubiquitinylated by Ube3A [16]. Sacsin’s N-terminus including SIRPT1 exhibited chaperoning activity in a luciferase folding assay, cooperatively with Hsp40 [12]. The sacsin J domain can bind Hsp70 chaperone family proteins and resolved aggregates of ataxin1 variants when overexpressed, which suggests that SacsJ acts as a co-chaperone [13]. Autophagy regulation is defective in SHSY5Y cells after knockout of the *SACS* gene and in fibroblasts derived from ARSACS patients [17], and autophagic flux was increased in patient-derived fibroblasts, although the mechanism by which sacsin is involved in autophagy is not known [18]. In fact, molecular chaperones, such as sacsin, associated with folding/unfolding and assembly/disassembly of protein complexes, have been considered as important players of selective autophagy [19]. 

A key feature of lack of sacsin is abnormal bundling of intermediate filaments (IF), including neurofilaments (NF) in *Sacs*^−/−^ mice and cultured neurons, and vimentin IF in cultured fibroblasts derived from patients’ skin biopsies [10,18]. Mitochondrial elongation is also a characteristic of cells lacking sacsin [3,20], although we showed mitochondrial abnormalities to be a later event than NF bundling in cultured *Sacs*^−/−^ motor neurons [10], and mitochondrial morphology in general to be dependent on a normal NF network [21]. We obtained direct evidence of sacsin’s function as a chaperone in IF assembly and homeostasis in cultured *Sacs*^−/−^ motor neurons and in patient-derived fibroblasts by ectopic expression of individual sacsin domains [22]. Subtle differences in the NF network were observed according to which of the sacsin domains was expressed: the UBL domain decreased NF bundles and the amount of NF proteins, without affecting NF assembly per se; SIRPT domains provided a scaffold for assembly of NF proteins into long filaments; the SacsJ domain prevented assembly of NF, and the HEPN domain re-localized filaments to the periphery of the cell, forming a cage-like structure [22]. 

IF are dynamic structures that undergo assembly/disassembly and annealing/severing. IF assembly is an organized stepwise process leading to the formation of 10 nm thick non-polar filaments [23]. IF proteins assemble into dimers through interaction of their coiled-coil domains, which then polymerize into tetramers. Eight tetramers assemble laterally to form a cylindrical Unit-Length Filaments (ULF) (~60 nm long), which anneal with other ULF to form an immature filament [24]. Radial compaction of the filament is the last step to form mature 10 nm diameter filaments, which can be solubilized in 8 M urea [25,26]. Although post-translational modifications play a regulatory role in IF assembly [27], so do a subset of chaperone proteins. For example, the small heat shock proteins HSPB1/Hsp25 and HSPB5/αB-crystallin decreased the rate of NFL polymerization and inhibited NFL transition from tetramers to filaments and their bundling in vitro [28]. HSPB5/αB-crystallin prevents IF assembly and network formation [29,30,31] and HSPB1/Hsp25 modifies the IF assembly dynamics, preventing extensive bundling of NF and maintaining an ordered NF assembly [32]. Finally, HSPA1A/Hsp70 can properly fold Charcot-Marie-Tooth type 2E-causing NFL variants into a normal filamentous network [21], but few co-chaperones like DNAJB6/Mrj have IF proteins as clients [33]. 

J-domain proteins, like Hsp40, commonly bind to unfolded proteins and recruit Hsp70 and other cofactors for refolding in an ATP-dependent manner [34]. The Sacs DNAJ domain reversed the thermosensitivity of a DNAJ-deficient *E. coli* [13]. We previously reported that expression of the SacsJ domain resolved NF bundles in motor neurons cultured from *Sacs*^−/−^ mice [22]. Compared to the UBL and SIRPT1 domains, SacsJ was the most effective in these neurons. In fibroblasts, SacsJ also promoted disassembly of the vimentin IF network [22]. Therefore, the questions that were addressed in this paper are: does SacsJ interact directly with IF proteins to affect assembly and does this require Hsp70 involvement. 

We tested whether a peptide corresponding to the SacsJ domain (aa 4316–4420) regulates IF assembly directly or indirectly, and whether it has a general chaperoning function on its own. In an in vitro NF assembly system, SacsJ both promoted dissolution of NF assembled from recombinant human NFL and inhibited their assembly, pointing to a direct role for this domain of sacsin in NF dynamics. We developed a cell-penetrating peptide to further examine SacsJ’s effect on IF in vivo. The cell-penetrating peptide SacsJ-myc-TAT significantly resolved NF bundles in cultured *Sacs*^−/−^ motor neurons, but also disassembled the IF network in *Sacs*^+/+^ fibroblasts and motor neurons. These data suggest that SacsJ is a potent regulator of IF networks, with implications for design of a sacsin mini gene for sacsin gene replacement therapy. 

## 2. Results

### 2.1. SacsJ Disassembled NFL Filaments In Vitro

**Figure 1:**
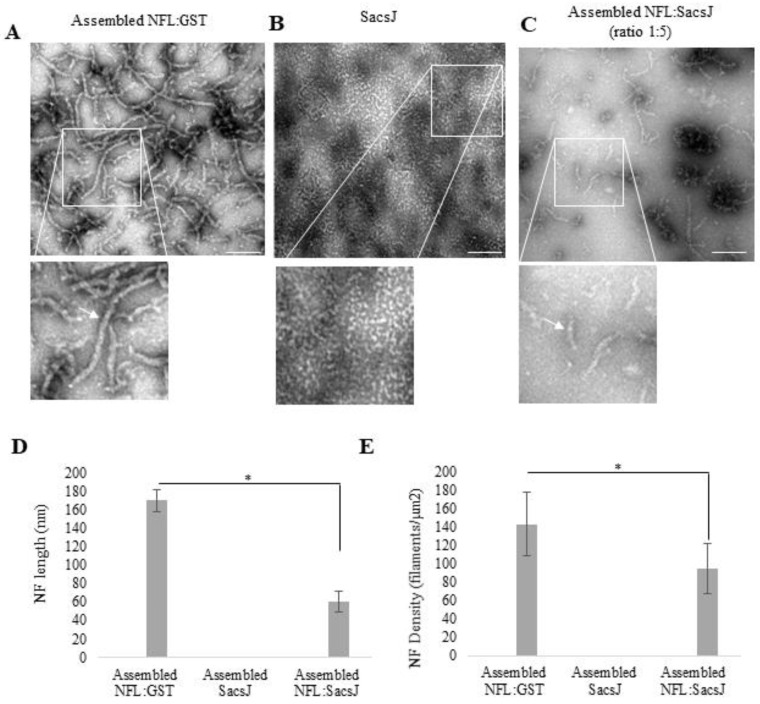
SacsJ disassembled NFL filaments in vitro. Shown are representative TEM images of filaments assembled from purified NFL (**A**). Assembled NFL filaments were incubated with or without SacsJ for 1 h at 37 °C and observed by TEM. Representative TEM images of filamentous NFL (**A**), SacsJ alone (**B**) or filamentous NFL co-incubated with SacsJ at a molar ratio of 1:5 (**C**). Inserts show enlargements of TEM images focused on NFL filaments (arrow). Scale Bar: 40 nm. Quantitation of NFL average length (**D**) and filament density (**E**) with or without incubation with SacsJ shows the significant decrease in NFL length and density (filaments/µm^2^). * *p* < 0.05 vs. NFL alone; one-way ANOVA, HSD Tuckey post hoc analysis (*n* = 3).

Ectopic expression of the SacsJ domain in *Sacs*^−/−^ motor neurons in culture resolved NF bundles [22]. To determine if the SacsJ domain can alter NF properties directly, we used an in vitro NF assembly assay. In vitro assembly of IF has been extensively studied and examination of the negatively stained filaments by transmission electron microscopy (TEM) is a standard method of characterization [35]. Purified recombinant human NFL incubated in assembly buffer formed filaments and some shorter filaments of 60 nm long that could correspond to ULF (Figure 1A); GST-SacsJ alone did not form filamentous structures following incubation in NFL assembly buffer (Figure 1B). After being assembled, NFL filaments were incubated with GST-SacsJ or GST as control. GST-SacsJ dismantled the pre-existing NFL filaments, as shown by the decreased average length and number of filaments observed by TEM (Figure 1C,E).

### 2.2. SacsJ Prevented In Vitro Assembly of NFL into Filaments

**Figure 2:**
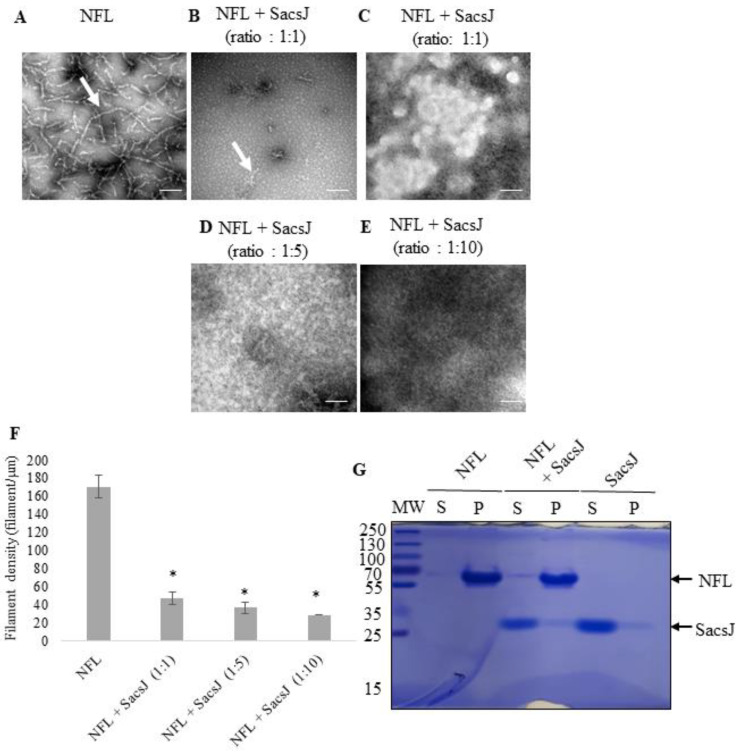
SacsJ impaired NFL assembly in vitro. (**A**–**E**) TEM of negatively stained preparations of assembled NFL dialyzed alone (**A**) or in the presence of different molar ratios of NFL to GST-SacsJ: 1:1 (**B**,**C**), 1:5 (**D**), and 1:10 (**E**). Scale bars: 100 nm. SacsJ prevented formation of NFL filaments (arrow). (**F**) Quantitation of NFL filament density: Incubation with GST-SacsJ significantly reduced filament density in a concentration-dependent fashion (filaments/µm^2^). * *p* < 0.05 vs. NFL alone, one-way ANOVA (*n* = 3). (**G**) SDS-PAGE of an in vitro sedimentation assay of NFL with or without SacsJ after assembly at 4 °C overnight at NFL:SacsJ ratio of 1:6. The supernatant (S) and pellet (P) fractions were analyzed by SDS-PAGE followed by Coomassie Blue staining. NFL and SacsJ bands are indicated by arrows and molecular weight is on the left.

To determine if SacsJ also prevents NF assembly, GST-SacsJ or GST were co-incubated with purified recombinant NFL in the NF assembly assay (Figure 2). Incubation of NFL with GST-SacsJ at a ratio of 1:1 (NFL:SacsJ) reduced the assembly of NFL into filaments (Figure 2B) as shown by the decrease of the average length of filamentous structures (Figure 2F). Instead, NFL formed large insoluble, amorphous aggregates consistent with the structure and assembly of NFL being disrupted (Figure 2C). Increased concentrations of GST-SacsJ at ratios of 5:1 and 10:1 totally prevented the formation of NFL filaments (Figure 2C–E). The average length of the NFL filaments was under 60 nm when GST-SacsJ was incubated with NFL during in vitro assembly, suggesting that the SacsJ domain prevented the assembly of NFL into ULF (Figure 2F). 

Interestingly, the small heat shock proteins HSPB1/Hsp25 or HSPB5/αB-crystallin are known to have such an effect on in vitro assembly of IF, releasing small dimers and tetramers in the soluble fraction of a sedimentation assay while filamentous IF were found in the pellet [28,30]. Therefore, the ability of SacsJ to change NFL sedimentation was assessed. After overnight incubation of NFL in assembly buffer (MES buffer) in vitro, with or without GST-SacsJ, supernatant and pellet fractions were separated by centrifugation and analyzed by SDS-PAGE; gels were stained with Coomassie blue to visualize protein bands (Figure 2G). Alone, NFL was mainly present in the pellet fraction (Pellet) and SacsJ in the supernatant fraction (Soluble), as expected from data presented in Figure 1. 

**Figure 3:**
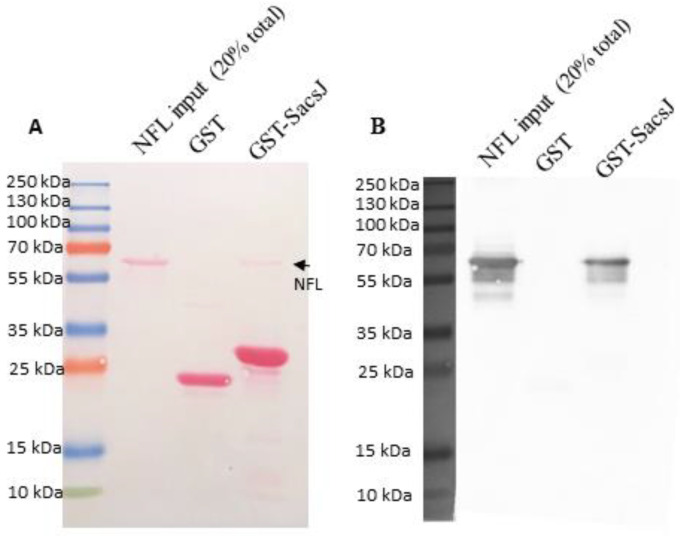
SacsJ directly interacts with NFL in vitro. (**A**) GST pulldown assay of soluble recombinant NFL with GST-SacsJ, or GST as control. (**A**) Ponceau red staining of the nitrocellulose membrane transferred from SDS-PAGE. Lanes are: NFL input—20% of total amount of NFL used in the pulldown assay; GST: GST incubated with NFL; GST-SacsJ: GST-SacsJ incubated with NFL. Bands stained by Ponceau red in each lane are NFL, GST, GST–SacsJ and NFL (small arrow). (**B**) Immunolabelling of the nitrocellulose membrane shown in (**A**) using anti-NFL antibody (NR4, 1/1000) demonstrating that the small band identified in (**A**) using GST-SacsJ as bait is indeed NFL.

That the solubility of NFL or SacsJ did not change despite obvious disruption of filament formation suggested they interact transiently rather than forming a stable complex. Also, the amount of soluble NFL in this assay is very low, rendering biochemical studies of protein interaction challenging. To better demonstrate a direct interaction between NFL and SacsJ, a GST pulldown assay was conducted using soluble recombinant NFL (see methods) and GST-SacsJ or GST as control (Figure 3A,B). Indeed GST-SacsJ, but not GST, pulled down NFL. By SDS-PAGE, a small band migrating at NFL’s molecular weight on SDS-PAGE was detected by Ponceau red staining when GST-SacsJ, but not GST, was used as bait. Immunolabelling by anti-NFL antibody confirmed that this band was NFL. Collectively these data suggest that SacsJ interacts with NFL to prevent the formation of filament and releases NFL to promote the formation of insoluble NFL aggregates. This is in contrast with HSPB1 which does move NFL into the soluble fraction [28]. 

### 2.3. The SacsJ Domain Lacks General Chaperoning Activity In Vitro

**Figure 4:**
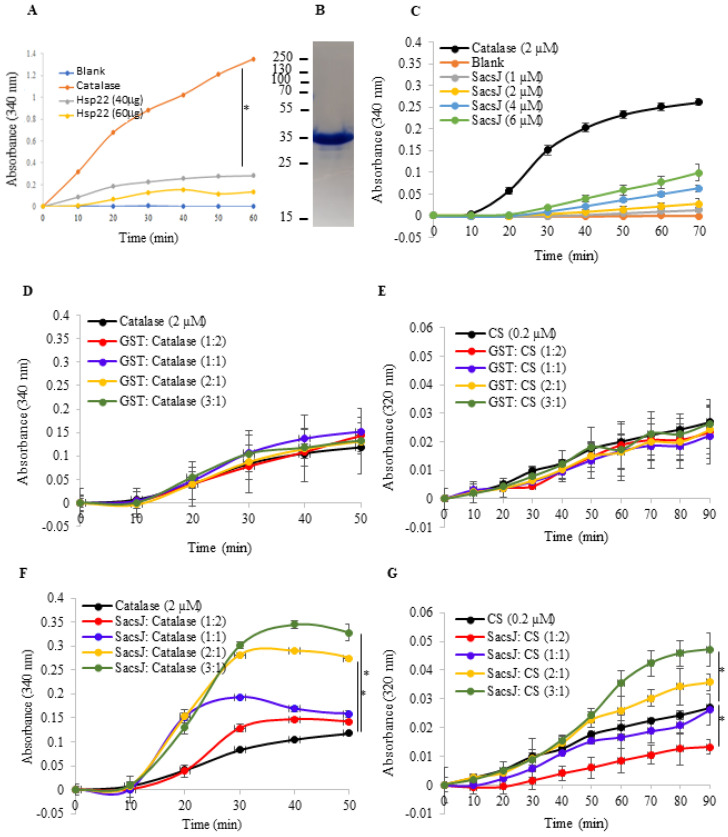
SacsJ domain did not act as a chaperone to reduce heat-denaturation of catalase or citrate synthase in an in vitro assay. (**A**) Absorbance at 340 nm of catalase (2 µM) incubated with or without Hsp22 used as a positive control (40 or 60 µg) heated at 60 °C. (**B**) Coomassie brilliant blue staining of a SDS-PAGE of purified recombinant SacsJ protein used in these assays. (**C**) Absorbance at 340 nm, as a function of time in an assay for heat-denaturation of catalase (2 µM) at 60 °C compared to heat-induced denaturation in the presence of increasing concentrations of SacsJ-GST (1–6 µM). (**D**,**E**) Absorbance at 340 nm of catalase (2 µM) or at 320 nm citrate synthase (CS) (0.2 µM) incubated with or without GST (1–6 µM) heated at 60 °C for catalase or 45 °C for CS. (**F**,**G**) Absorbance at 340 nm and 320 nm of catalase (2 µM) or citrate synthase (CS) (0.2 µM) incubated with or without SacsJ-GST (1–6 µM) heated at 60 °C for catalase or 45 °C for CS. * *p* < 0.05 vs. catalase or CS alone, one-way ANOVA, HSD Tuckey post hoc analysis (*n* = 3).

Since the SacsJ domain alone altered NFL assembly in vitro, we tested whether this effect was specific or if SacsJ could function as a protein chaperone more generally. This was tested using an in vitro chaperoning assay based on heat-induced denaturation of catalase or citrate synthase (CS) [36]. Following heat-exposure, substrates such as CS and catalase unfold and form aggregates, measured by increased spectrophotometric absorbance at 320 nm or 340 nm, respectively; chaperoning activity counteracts substrate aggregation and thus the increase in absorbance in the assay. Purified CS and catalase denature at different temperatures, 45 °C and 60 °C within 90 or 60 min, respectively. In order to assess if SacsJ would prevent aggregation of substrates, GST (Figure 4C,D) or GST-SacsJ (Figure 4E,F) were mixed with catalase or CS at different molar ratios in the heat denaturation assay. At the molar ratio of 1:1 SacsJ:catalase, GST-SacsJ did not reduce the aggregation of catalase (Figure 4E). Rather, increasing concentrations of GST-SacsJ exacerbated the aggregation of catalase, suggesting that SacsJ either aggregates itself or promotes unfolding and aggregation of the substrate. When tested alone in the assay, GST-SacsJ aggregated to some extent in a concentration-dependent fashion, but far below levels achieved by catalase (Figure 4B); i.e., the increased absorbance obtained by combining catalase with SacsJ was much greater than the sum of absorbance measured individually. With CS as the substrate, SacsJ reduced its aggregation only at a molar ratio of 1:2 SacsJ:CS (Figure 4F); the higher concentrations of GST-SacsJ increased CS aggregation (molar ratios 2:1 and 3:1), similar to experiments with catalase. All together, the data confirm that SacsJ alone lacks significant inherent, general chaperoning activity, suggesting that the SacsJ effect on NFL formation is due to specific disruption of NF structure.

### 2.4. A Cell-Permeant SacsJ Peptide Disassembld Vimentin Filaments in Fibroblasts 

**Figure 5:**
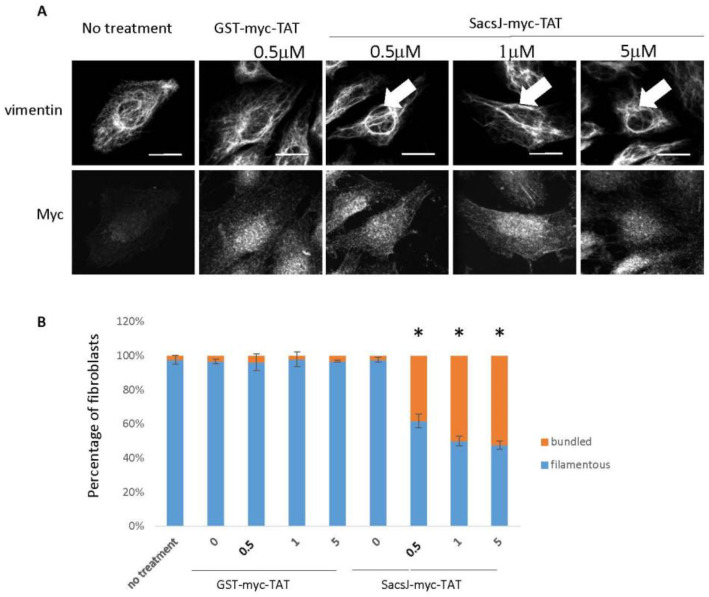
The cell-permeant peptide, Sacs-myc-TAT, induced step-wise disruption of the vimentin IF network in MCH74 fibroblasts. (**A**) Representative 3-dimensional reconstructions of Z-stack confocal images of MCH74 fibroblasts double labelled with anti-myc (rabbit anti-myc) and anti-vimentin (V9 mouse monoclonal) treated with increasing concentrations of Sacs-myc-TAT as indicated (0.5 to 5 µM) or control peptide GST-myc-TAT. Treatment with Sacs-myc-TAT for 30 min resulted in nuclear rings of bundled vimentin IF (large arrow). Scale bar: 20 µm. (**B**) Quantitation of the percentage of fibroblasts presenting circum-nuclear IF bundles or finely distributed IF when treated with increasing concentrations of Sacs-myc-TAT (0.5 to 5 µM) showing an increase in the percentage of cells with IF concentrated surrounding the nucleus. * *p* < 0.05 vs. NFL alone, one-way ANOVA (*n* = 3). Note the diffuse distribution of labelling of the myc tag on SacsJ.

Our previous studies in cultured fibroblasts and motor neurons used plasmid delivery of myc-tagged sacsin domains [22], which is difficult to administer to neurons and other primary cells with low transfectability. We therefore developed a cell-permeant peptide (SacsJ-myc-TAT) in which the amino acid sequence of the SacsJ domain was fused to a myc epitope for detection and a cell penetrating sequence from the trans-activating transcriptional activator of HIV1 (TAT) for delivery into cells. GST-myc-TAT was also generated as a control. An immortalized line of the normal primary fibroblast strain MCH74 was used to establish dose-response for intracellular delivery of the peptide and its effect on vimentin IF. Fibroblasts were treated with 0.5 µM, 1 µM or 5 µM SacsJ-myc-TAT or GST-myc-TAT for 30 min, and the intracellular uptake of these peptides into the fibroblasts was detected by double label immunocytochemistry using antibodies against the myc-tag and vimentin. As shown in the confocal micrographs in Figure 4A, both peptides were detected in the cytoplasm and to a lesser extend in the nuclei of fibroblasts after 30 min, which is consistent with the biochemical properties of the TAT sequence [37,38]. Fibroblasts receiving no treatment served as a control. Interestingly, the formation of perinuclear rings of vimentin (Figure 5A) was a phenotype observed in fibroblasts treated with the SacsJ-myc-TAT for this short time period. At 0.5 µM the percentage of fibroblasts showing this phenotype was 38 ± 2.5% compared to 3.8 ± 1.3% in cultures treated with GST-myc-TAT and 2.3 ± 2.5% with no treatment (Figure 5B). This percentage was only slightly increased at higher concentrations of SacsJ-myc-TAT (1 or 5 µM) (Figure 5B). 

**Figure 6:**
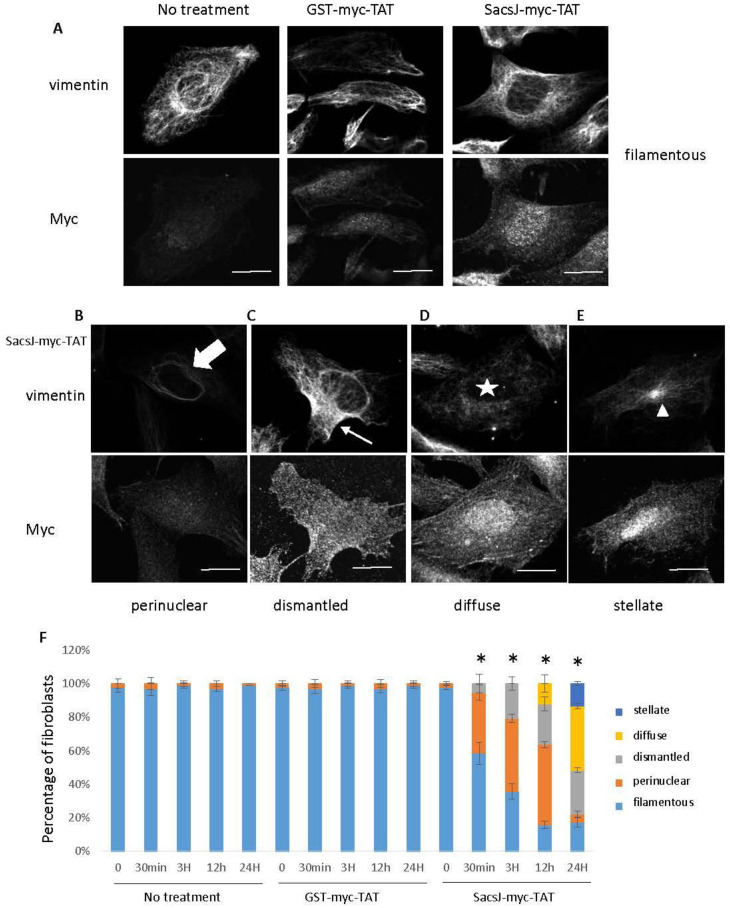
Over time, SacsJ-myc-TAT treatment resulted in the disassembly of vimentin IF in normal *Sacs*^+/+^ (MCH74) fibroblasts. (**A**) Representative 3-dimensional reconstructions of Z-stack confocal images of fibroblasts double labelled with anti-myc (rabbit anti-myc) and anti-vimentin (V9 mouse monoclonal). Scale bar: 20 µm. (**B**–**E**) MCH74 fibroblasts were treated with GST-myc-TAT control or SacsJ-myc-TAT (0.5 µM) for 30 min, 3 h, 12 h or 24 h and showed time-dependent phenotypes: perinuclear rings of vimentin ((**B**), large arrow), dismantled vimentin network ((**C**), small arrow), diffuse vimentin labelling ((**D**), star), and appearance at a stellate accumulation ((**E**), arrowhead). (**F**) Quantitation of the percentage of fibroblasts presenting those phenotypes over duration of SacsJ-myc-TAT treatment. * *p* < 0.05 vs. time-matched no treatment or treated with GST-myc-TAT (0.5 µM) one-way ANOVA, HSD Tuckey post hoc analysis (*n* = 3).

Next, the effect of longer treatment durations with the SacsJ-myc-TAT or the GST-myc-TAT on the vimentin network was tested. Fibroblast cultures were treated with 0.5 µM SacsJ-myc-TAT or GST-myc-TAT for 0 min, 30 min, 3 h, 12 h or 24 h, followed by double immunolabeling for vimentin and the myc tag (Figure 6). The GST-myc-TAT was detected intracellularly by indirect immunofluorescence up to 24 h suggesting a certain stability while, the SacsJ-myc-TAT was barely detectable at 24 h. Qualitatively, the vimentin network appeared as a classic filamentous network in untreated fibroblasts or GST-myc-TAT-treated fibroblasts at all incubation times (Figure 6A). On the other hand, fibroblasts treated with SacsJ-myc-TAT presented time-dependent phenotypes (illustrated in Figure 6A,B). Perinuclear rings vimentin were observed at the 30 min time point as described above (Figure 6B), but at 3 h, the vimentin network was dismantling (Figure 6C). By 12 h, cells showed diffuse, barely detectable, labelling of vimentin (Figure 6D). At 24 h, a juxtanuclear focus of vimentin labelling was observed (stellate in Figure 6E), which together with a barely detectable SacsJ-myc-TAT suggests that this phenotype represents a recovery stage. 

The perinuclear collapse and reappearance of vimentin IF is consistent with their normal process of organization, which is dependent on interaction with the LINC complex (linker of the nucleocytoskeleton and cytoskeleton) at the nucleus, extension along microtubules and interaction with plasma membrane proteins [39]. Fall back of IF to a perinuclear/juxtanuclear position, such as occurs with anti-microtubule agents [40,41,42], likely represents a stage of dissolution of the IF network rather than an active effect of SacsJ. As shown in Figure 6F, the percentage of fibroblasts presenting perinuclear vimentin remained relatively constant during the first 12 h in the range of 36 ± 6.5% to 44 ± 5.75% (at 30 min and 12 h of treatment, respectively) with no statistical difference by one-way ANOVA and a Tuckey HSD post hoc analysis. Similarly, the percentage of fibroblasts with a dismantled vimentin network remained constant from 3 h (20.64 ± 4%) to 24 h (26.16 ± 1.43%) while the percentage of fibroblasts with juxtanuclear, stellate vimentin (13.51 ± 1.3%), which represents recovery, increased. All together, our data suggest a dynamic regulation of the vimentin network by the SacsJ-myc-TAT leading to the stepwise disassembly of the vimentin network, which was captured by this experiment. Importantly, the SacsJ-myc-TAT peptide was taken up by the fibroblasts and reproduced the effect of the plasmid-derived ectopic expression of the SacsJ domain in disassembling the vimentin network [22]. 

### 2.5. SacsJ Disassembled NF in Mouse Motor Neurons in Culture 

**Figure 7:**
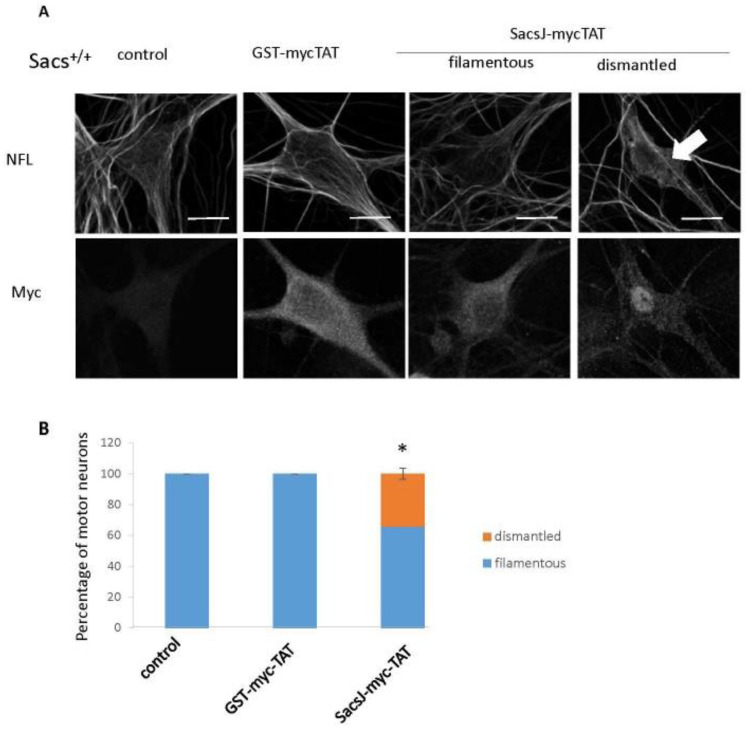
The cell-permeant peptide, SacsJ-myc-TAT, induced the disassembly of NF in *Sacs*^+/+^ motor neuron in culture. (**A**) Representative 3-dimensional reconstructions of Z-stack confocal images of double labelling with anti-myc (rabbit anti-myc) and anti-NFL in *Sacs*^+/+^ 6 week-old murine spinal cord-DRG cultures showing the NF network and distribution of myc-TAT peptides in motor neurons. Cultures were treated with SacsJ-myc-TAT (0.5 µM) or GST-myc-TAT control peptide for 30 min and compared to untreated cultures. SacsJ-myc-TAT dismantled the endogenous NF network (large arrow). Scale bar: 20 μm. (**B**) Quantitation of the percentage of motor neurons presenting a filamentous or dismantled NF network when treated with Sacs-myc-TAT (0.5 μM). * *p* < 0.05 vs. no treatment or treated with GST-myc-TAT (0.5 µM) using a one-way ANOVA, HSD Tuckey post hoc analysis (*n* = 3).

**Figure 8:**
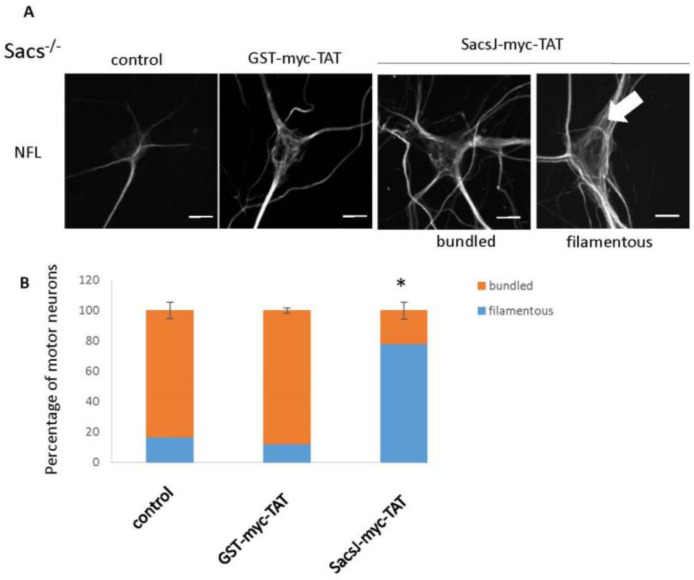
The cell-penetrating peptide, SacsJ-myc-TAT, resolved NFL bundles in *Sacs*^−/−^ motor neuron in culture. (**A**) Representative 3-dimensional reconstructions of Z-stack confocal images of motor neurons in *Sacs*^−/−^ 6 week-old spinal cord-DRG cultures double labelled with anti-myc (rabbit anti-myc) and anti-NFL to show the NF network and SacsJ-myc-TAT distribution in motor neurons. Cultures were treated with SacsJ-myc-TAT (0.5 µM) or GST-myc-TAT control peptide for 30 min and compared to untreated cultures. Treatment with SacsJ-myc-TAT resolved the NF bundles (large arrow). Scale bar: 20 µm. (**B**) Quantitation of the percentage of motor neurons presenting a filamentous or bundled NF network when treated with Sacs-myc-TAT (0.5 µM). * *p* < 0.05 vs. no treatment or treated with GST-myc-TAT (0.5 µM) using a one-way ANOVA, HSD Tuckey post hoc analysis (*n* = 3).

To determine the efficiency of SacsJ-Myc-TAT to resolve the NF network and bundles in sacsin wild-type and sascin-deficient neurons, motor neurons in 6 week-old *Sacs*^+/+^ or *Sacs*^−/−^ spinal cord-DRG cultures were treated with 0.5 µM SacsJ-Myc-TAT or its control, GST-myc-TAT, for 30 min. Intracellular uptake of SacsJ-Myc-TAT or GST-myc-TAT was detected by anti-myc immunolabeling and the NF network was visualized and evaluated by double-labelling cells with anti-NFL (Figure 7A and Figure 8A). Qualitatively, the NF network in *Sacs*^+/+^ motor neurons either remained filamentous or was dismantled/disassembled (Figure 7A,B) in cultures treated with the SacsJ-Myc-TAT peptide. In 6 week-old *Sacs*^−/−^ spinal cord-DRG cultures, 83 ± 5% of motor neurons contained well-established NF bundles (Figure 8A), which decreased to 22.08 ± 5% of neurons following treatment with SacsJ-myc-TAT; GST-myc-TAT had no significant effect (Figure 8B). Qualitatively, the NF networks in *Sacs*^−/−^ motor neurons treated with GST-myc-TAT were more spatially distributed rather than bundled in dendrites and cell bodies. 

**Figure 9:**
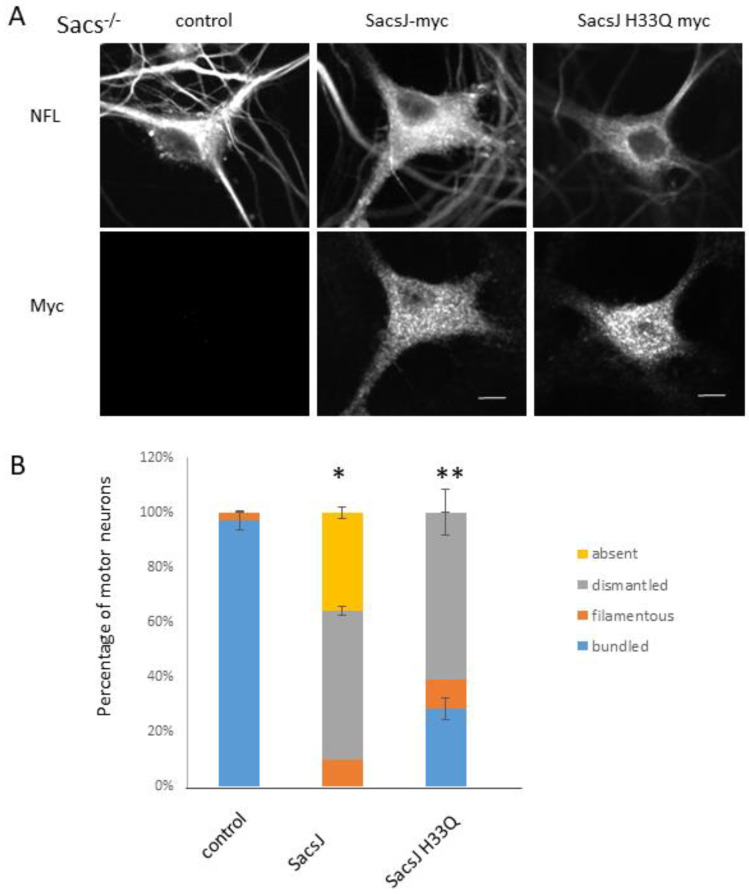
SacsJ acts independently to disassemble NF, but requires Hsp70 to degrade NFL. SacsJ-myc or SacsJH33Q-myc, a variant unable to bind HSP70, were expressed in motor neurons in *Sacs*^−/−^ 6 week-old spinal cord-DRG cultures. (**A**) Representative 3-dimensional reconstructions of Z-stack confocal images of motor neurons double labelled with anti-myc (rabbit anti-myc) and anti-NFL to show the NF network and expression of NFL. The NF network was dismantled or absent when SacsJ-myc was expressed while SacsJH33Q-myc only dismantled NF network. Scale bar: 10 µm. (**B**) Quantitation of the percentage of motor neurons presenting a filamentous, bundled, dismantled or no NF network when expressing SacsJ-myc or SacsJH33Q-myc. * *p* < 0.05 vs. control, ** *p* < 0.05 vs. SacsJ using a one-way ANOVA, HSD Tuckey post hoc analysis (*n* = 3).

The experiments in vitro indicated a direct role for SacsJ in assembly/disassembly of NFL filaments, but not general chaperoning activity preventing thermal denaturation. Yet, either ectopic expression of Hsp70 or induction by celastrol resolved NF bundling in *Sacs*^−/−^ motor neurons in culture [22]. In order to determine the respective role of Hsp70 and of the SacsJ domain in resolving NF bundles, the histidine of the conserved HPD sequence of J-domains known to be responsible of Hsp70 binding was mutated to generate the SacsJH33Q variant defective in binding Hsp70 (Figure S3A) as in [13]. Again, NF bundles were resolved by the expression of the SacsJ-myc; the NF network was either dismantled into unassembled NFL, indicated by the dotted labeling pattern, or was undetected by immunolabeling in respectively 54.45 ± 5% and 35 ± 2.6% of cultured *Sacs*^−/−^ motor neurons expressing SacsJ-myc (Figure 9). The expression of the SacsJH33Q-myc variant also dismantled NF bundles into a mix of thin filaments and unassembled NFL, although the depletion that occurred in some neurons expressing SacsJ-myc was not observed (Figure 9B). Thus, SacsJ can disrupt assembled NF independent of Hsp70, but interaction with Hsp70 may be required for the further degradation of endogenous NFL. Indeed, linear regression analysis showed a negative relationship between expression levels of SacsJ-myc and NFL (Figure S3, R2 = 0.6368), while there was no correlation between the expression levels of SacsJH33Q-myc and NFL (Figure S3, R2 = 0.0445). It is not to exclude that the effect of SacsJH33Q on NFL network could be due to the overexpression of ectopic SacsJH33Q. Indeed, overexpression of SacsJH33Q relative to NF proteins could interfere with normal assembly of NF proteins by preventing the formation and assembly of NF dimers or high order oligomers in this non-physiological condition. Unfortunately, our experimental cell model does not allow biochemical analysis to demonstrate the formation of NF oligomers by BN-PAGE following the ectopic expression of SacsJH33Q or SacsJ. 

## 3. Discussion

This study demonstrated a direct role of the SacsJ domain of sacsin in IF assembly/disassembly. In vitro, SacsJ disrupted both pre-existing NFL filaments and the formation of new NFL filaments, in the absence of any Hsp70. In cellular models, the cell-penetrating peptide SacsJ-myc-TAT disrupted the vimentin IF network in fibroblasts and the NF network in cultured motor neurons. The peptide was even more efficient in clearing the abnormal bundles of NF in *Sacs*^−/−^ motor neurons. These results suggest that sacsin through the J-domain is a potent regulator of IF networks. and joins the few chaperone proteins known to regulate IF homeostasis. 

While assembly of IF proteins is mainly regulated by phosphorylation of N-terminal residues [27], some small heat shock protein chaperones, HSPB1 and HSPB6, can prevent the assembly of IF, disassemble IF into dimers and tetramers, and maintain IF proteins soluble in non-denaturing detergent [28,30,43]. In contrast to HSPB1 or HSPB6, the SacsJ domain did not solubilize or co-sediment with NFL in vitro, despite the fact that NFL filaments were dismantled or prevented from assembling. When incubated with the SacsJ domain, NFL pelleted following centrifugation rather than being retained in the supernatant as would dimers and tetramers [30], but the absence of intact filaments and shorter filaments corresponding to ULF by TEM indicates the presence of some intermediate sized form of NFL in addition to aggregates in the presence of SacsJ. 

This study demonstrated the interrelationship between the J domain of Sacsin with IF. This finding is reminiscent of DNAJB6/MrJ, which interacts directly with keratin K8/K18. A role of DNAJB6 in keratin disassembly is supported by the abnormal bundling of filaments following intracellular microinjection of a neutralizing antibody [33]. In fact, Hsp40 has been shown to play a role in the exchange of the Keratin5/Keratin14 pair to the Keratin1/Keratin10 pair into the keratin filaments and to regulate keratin proteins via the ubiquitin-proteasome pathway, with the participation of Hsc70 [33,44]. Our data point to a similar capability of the SacsJ domain of sacsin and Hsp70. While ectopic expression of SacsJ in cultured motor neurons resulted in the dismantling of NF bundles and in the loss of NFL immunolabeling, the expression of the SacsJH33Q variant lacking Hsp70 binding ability dismantled NF bundles in cultured *Sacs*^−/−^ motor neurons into thin filaments and unassembled NFL, without affecting NFL expression levels. 

We also developed a cell-penetrating peptide, SacsJ-myc-TAT, that retained SacsJ properties identified previously by plasmid-mediated delivery [22], specifically, disassembly of the vimentin IF network in fibroblasts. The peptide delivery system facilitated examination of the effects of SacsJ on the network over time in vivo and revealed a stepwise disorganization of vimentin IF, starting by depletion of IF in the periphery and concentrating around the nucleus and progressing to full dissolution and loss of vimentin labeling. The filaments around the nucleus are reminiscent of the perinuclear rings of vimentin, described when microtubules are disrupted such as by treatment with colcemid [40,41,42] or inhibition of PP2A [39]; i.e., they are first to form and last to go [18,45]. In addition, we showed that turnover of subunits is slower in bundled NF in *Sacs*^−/−^ motor neurons in culture [22]. 

The cell-penetrating peptide SacsJ-myc-TAT was also tested in spinal cord-DRG cultures, resulting in dissolution of NF in motor neurons. In cultures prepared from *Sacs*^−/−^ mice, SacsJ-myc-TAT resolved the characteristic NF bundles characteristic of ARSACS. The challenge with sacsin replacement therapy is that the sequence is too large for delivery methods. Whereas a more normal NF network was preserved in *Sacs*^−/−^ neurons treated with the SacsJ peptide, the loss of IF networks in *Sacs*^+/+^ fibroblasts or neurons indicate that use of the SacsJ domain alone therapeutically could be problematic over the longer term or dosage would be crucial. This study points to the important role the J-domain of sacsin would play in IF remodeling and its importance as a component of a sacsin mini gene.

## 4. Materials and Methods

### 4.1. Cloning, Protein Production and Purification

The SacsJ cDNA (corresponding to residues 4316–4420) inserted in frame with GST into the pGEX6 vector was amplified from the mouse pEGFP-sacsin full length (OriGene Technologies, Rockville, MD, USA). For delivery into cells and tissues, the pGEX-SacsJ or GST were tagged on the C-terminus with a myc epitope to identify the peptide and with the TAT-derived cell- penetrating peptide sequence (YGRKKRRQRRR) (36), which has been shown to confer efficient neuronal delivery and blood-brain barrier penetration (37). The H33Q mutation was introduced into pcDNA4.1-SacsJ-myc in order to produce a J-domain variant defective in binding Hsp70. Constructs are described in Figure S2. All cloning was subcontracted to NorClone (London, ON, Canada, NorClone—Gene Cloning Supplier). Human NFL cloned into the pET23b expression vector was a gift of Dr. Walter Mushynski (retired, McGill University). 

For recombinant protein production, plasmids carrying the SacsJ-myc-TAT or GST-myc-TAT cDNA were transformed into the Escherichia coli BL21 (DE3) pLysS. Bacterial cultures were grown overnight at 37 °C and then diluted at 1:100 (*v*/*v*) into 1 L Luria–Bertani medium containing 100 μg/mL ampicillin. Bacteria were grown at 37 °C with vigorous shaking at 225 rpm until they reach an OD600 nm = 0.6, recombinant protein expression was then induced using 1 mM isopropyl-1-thio-β-D galactopyranoside (IPTG) at 30 °C for 4 h.

Bacteria were lysed in PBS buffer (137 mM NaCl, 2.7 mM KCl, 10 mM Na_2_HPO_4_, 2 mM KH_2_PO_4_ pH 7.4), supplemented with 1 mM phenylmethylsulfonyl fluoride (PMSF), 80 units of DNase,100 µg/mL lysozyme and Triton 0.1%. After sonication, bacterial debris was pelleted by centrifugation. The supernatant was then filtered through a 0.2 µm filter and chromatography affinity purification of GST-myc-TAT or GST-SacsJ-myc-TAT using glutathione resin was carried out according to the manufacturer instructions (GE-Healthcare, Mississauga, ON, Canada). 

Production of recombinant human NFL was induced in the presence of 0.4 mM IPTG for 4 h at 37 °C according to Leung and Liem [35]. Briefly, after bacteria lysis, NFL was recovered in inclusion bodies. The insoluble protein pellet containing NFL was solubilised in Buffer 1 (8 M urea, 10 mM sodium phosphate buffer, pH 7.4, 0.1% (*v*/*v*) 2-mercaptoethanol and 1× proteasome inhibitors (Sigma-Aldrich, Oakville, ON, Canada) and centrifuged. The supernatant was filtered through a 0.45-µm syringe filters and solubilized NFL was then purified from the supernatant by affinity chromatography using Bio-gel^®^ HT hydroxyapatite (130-0150, Bio-Rad, Mississauga, ON, Canada) and following the protocol of Leung and Liem [35]. The column was then washed with Buffer 2 (8 M urea, 100 mM sodium phosphate buffer, pH 7, 0.1% (*v*/*v*) 2-mercaptoethanol and 1× proteases inhibitors) and NFL was eluted in Buffer 3 (8 M urea, 300 mM sodium phosphate buffer, pH 7, 0.1% (*v*/*v*) 2-mercaptoethanol and 1× proteases inhibitors).

### 4.2. In Vitro Assembly of NFL and Pulldown Assay 

The assembly of NFL followed the procedure of Leung and Liem [35]. Urea was gradually removed by dialysis against the assembly buffer (50 mM MES, 0.5 mM EGTA, 0.175 M NaCl, 1 mM DTT, 0.4 mM PMSF, pH 6.8) containing 4 M urea, 2 M urea, 1 M urea or no urea using a D-Tube Dialyzer MWCO 6–8 kDa (MilliporeSigma, Oakville, ON, Canada), to favor a proper assembly. In these conditions, IF proteins sequentially assemble into dimers, tetramers and filamentous structures rather than non-specific aggregates (Figure S1) [46]. To study the effect of SacsJ on NFL assembly, SacsJ was added to the assembly buffer without urea within the first 10 min of dialysis when NFL are not assembled yet into a filamentous structure [47]. NFL assembly (0.24 µg/µL total) was achieved by overnight dialysis at 4 °C. Filament assembly was assessed by the formation of filamentous structures observed by electron microscopy and by biochemical analysis using the insoluble properties of assembled NFL. After overnight dialysis, the solution containing assembled NFL was incubated with or without GST-SacsJ for 12 h and then centrifuged at 100,000× *g* for 30 min at 20 °C to separate the soluble and insoluble fractions, containing respectively soluble NFL and dimers or filamentous NFL. The soluble and insoluble fractions were then analyzed by SDS-PAGE using Coomassie Blue staining. Soluble NFL, obtained as described above, was incubated with GST or GST-SacsJ, as baits, during 2 h in assembly buffer before 100 μL of glutathione beads equilibrated in assembly buffer were added and incubated during 1 h. Baits and protein complexes bound to the glutathione beads were then pulled-down by centrifugation at 13,000× *g* during 1 min, and washed 3 times with assembly buffer. Samples were then analyzed by SDS-PAGE and electrotransfered onto a nitrocellulose membrane for immunoblotting using anti-NFL antibody (1:1000 clone NR4, Sigma-Aldrich). 

### 4.3. Negative Staining and Electron Microscopy

Electron microscopy was performed at the Facility for Electron Microscopy Research (FEMR) at McGill University. 10 µL of protein samples were deposited and adsorbed for 1 min onto carbon-coated grids that were glow discharged before the application of proteins. Grids were glow discharged for 1–2 min in a vacuum evaporator (Edwards Vacuum Carbon Coater E306) before adding the protein samples to improve quality of sample analysis. Assembled NFL as well as SacsJ were diluted to 0.24 µg/µL and processed for imaging by TEM. Excess protein was removed by wicking the edge of the grid on a piece of Whatman paper. The samples were then negatively stained for 1 min using 10 µL of 2% (*w*/*v*) uranyl acetate and examined with a Cryo-TEM (FEI Tecnai G2 Spirit Twin) (Thermo Fisher Scientific, St Laurent, QC, Canada) using an accelerated voltage of 120 KV. Images were acquired with a CCD camera (Gatan Ultrascan 4000 4 k × 4 k CCD Camera System Model 895). The diameters of the assembled filaments were measured on enlarged TEM micrographs using ImageJ software (National Institute of Health, Bethesda, MD, USA; https://imagej.nih.gov/ij/ accessed on 1 May 2020).

### 4.4. Cell Culture

Human skin fibroblasts were from the CellBank Repository for Mutant Human Cell Strains (McGill University Health Complex, Montreal, QC, Canada). Immortalised fibroblasts were cultured in Dulbecco’s Modified Essential Medium with 10% fetal bovine serum (FBS). Cultures were treated were treated with GST-myc-TAT or GST-SacsJ-myc-TAT to assess the time-dependence (30 min to 24 h) and dose-dependence (0–5 µM) of the peptides’ effects on the vimentin IF network, visualized by immunolabeling with antibody to vimentin (clone V9, MA5-11883 Thermo Fisher, St Laurent, QC, Canada). Peptides were identified by immunolabeling using an anti-myc antibody (C3956, Sigma-Aldrich, Oakville, ON, Canada). Cy2 or Cy3 conjugated donkey secondary antibodies against mouse or rabbit IgG were from Jackson ImmunoResearch (1/300). 

Primary cultures of dissociated spinal cord-dorsal root ganglia (DRG) were prepared from E13 *Sacs*^−/−^ mice (C57Bl6 background) and wild type (*Sacs*^+/+^) of the same background. Generation and characterization of the *Sacs*^−/−^ mice were as previously described [10]. Cells were plated on glass coverslips (Fisher, Toronto, ON, Canada) coated with poly-D-lysine (P7280, Sigma-Aldrich) and Matrigel^®^ (CACB354234, VWR, Mississauga, ON, Canada) and maintained in Eagle’s Minimum Essential Medium enriched with 5 g/l glucose and supplemented with 3% horse serum, and other growth factors as previously described [48]. Cultures were used in experiments 6 weeks following plating to allow neuronal maturation and appearance of NF bundles in more than 80% of *Sacs*^−/−^ motor neurons. NF were visualized by immunolabeling with anti-NFL (clone NR4, N5139, Sigma-Aldrich). NF bundles were defined as previously by a continuous compact filament bundle crossing the cell body from dendrite to dendrite, distinguished from the normal finer NF network [22]. Because motor neurons in long-term primary spinal cord cultures are not transfectable, plasmids encoding SacsJ-myc or SacsJH33Q-myc were expressed by intranuclear microinjection of pcDNA4.1-SacsJ-myc and pcDNA4.1-SacsJH33Q-myc (at 20 μg/mL) in *Sacs*^−/−^ motor neurons in culture as described in (23). Forty-eight hours after injection, indirect immunofluorescence detection of NFL and ectopic SacsJ or SacsJH33Q was used to assess the expression levels; fluorescence intensity was measured using ImageJ and regression analysis was carried out using excel. 

### 4.5. In Vitro Chaperone Assay

Chaperone activity of GST-SacsJ was determined in a heat-induced aggregation assay, measuring its ability to prevent heat denaturation of two protein substrates: pig citrate synthase (CS) (0.2 μM; MW 51 kDa; Sigma-Aldrich, Oakville, ON, Canada), and catalase purified from bovine liver (2 µM; MW 250 kDa; Sigma-Aldrich, Oakville, ON, Canada). Time duration for this assay was 90 min for CS and 50 min for catalase. Absorbance was measured every 10 min at 320 nm or 340 nm for CS or catalase, respectively. The substrates were heat-denatured at 45 °C for CS and at 60 °C for catalase in the absence or presence of SacsJ equal to 0.1–0.6 μM for CS and 1–6 μM for catalase. All assays were performed in 96-well clear-bottom plates (Sarstedt, Montreal, QC, Canada) using a spectrophotometer with thermostated cells (Varian Cary 100, Montreal, QC, Canada) in HEPES buffer (50 mM HEPES-KOH, pH 7.5) for CS and Sodium Phosphate buffer (100 mM, pH 7.5) for catalase [49]. Data are representative of three independent assays and are expressed as the mean ± SD.

### 4.6. Statistics

Quantitative experiments are presented as mean ± SD. Length and number of NFL filaments were measured on TEM images taken from at least two separate dialysis batches. The proportion of motor neurons or fibroblasts carrying a dismantled (i.e., shorter filaments and absence of a continuous network), regular (i.e., continuous network) or bundled IF network was quantified in at least three coverslips per condition, with a minimum count of 30 cells per coverslip. *p* values < 0.05 were considered statistically significant. Statistical analysis was performed by using one-way ANOVA followed by a Tuckey HSD post hoc-analysis.

### 4.7. Ethics Statement

Experiments were approved by the Animal Care Committee of the Montreal Neurological Institute (McGill University protocol #7437) and all procedures followed the Canadian Council on Animal Care guidelines. Secondary use of human-derived cells was approved by McGill ethic committee (A07-M34-19B).

## Data Availability

The data presented in this study are available from the corresponding author upon reasonable request.

## Supplementary Materials

The following supporting information can be downloaded at: https://www.mdpi.com/article/10.3390/ijms232415742/s1. References [11,14] are cited in the Supplementary Materials.
