# Supplementary material for: The J Domain of Sacsin Disrupts Intermediate Filament Assembly
Source: Int J Mol Sci. 2022 Dec 12;23(24):15742. doi: 10.3390/ijms232415742 (PMC9779362; doi:10.3390/ijms232415742)
Supplement: Supplementary file 1 [file ijms-23-15742-s001.zip › ijms-1962958-supplementary.pdf]

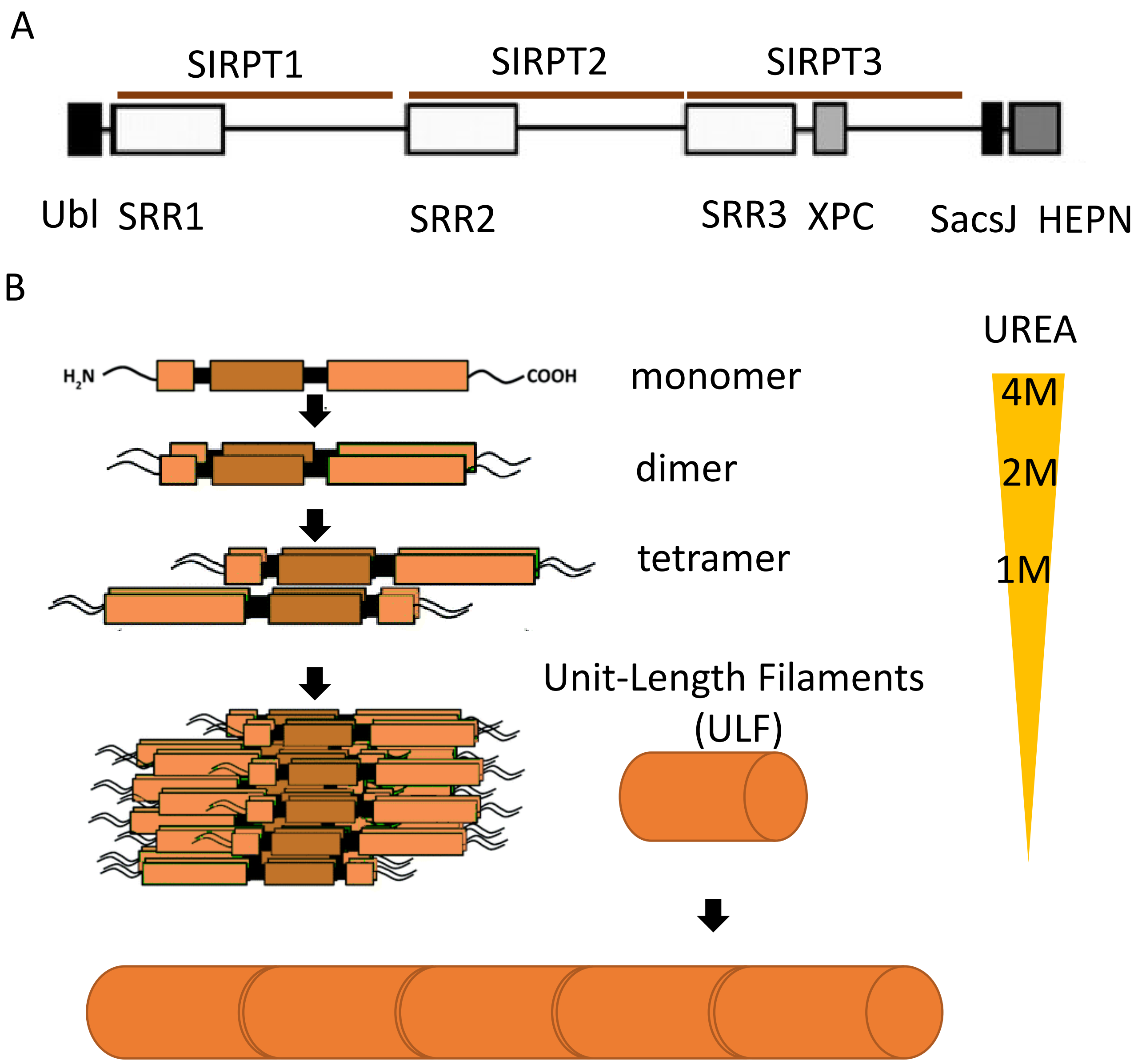

Fig S1 Supplemental. (A) Putative domains in sacsin protein (aa 1-4579). From N-terminus to the C-terminus: ubiquitin-like (Ubl aa 1-80; PFAM acc. no. PF00240), Sacsin *Internal RePeaTs* (*SIRPTs*) as defined by Romano *et al*. [11] containing histidine kinase-like ATPase (HATPase in orange) and the sacsin repeating region (SRR) supradomains *SIRPT1* (amino acids 84 – 1,374), *SIRPT2* (aa 1,444–2,443), *SIRPT3* (aa 2,512–3,896); the putative sacsin Xeroderma Pigmentosum group C. Binding domain (XPCB aa 3698-3736); DnaJ (aa 4316-4366); molecular chaperone homology domain; SMART acc. no. SM00271), HEPN (aa 4447-4574; higher eukaryotes and prokaryotes nucleotide-binding domain; SMART acc. no. SM00748). Adapted from Anderson *et al*. [14] **(B) Illustration of the sequential assembly of intermediate filaments from monomers to filamentous structures.** Intermediate filaments arise from dimerization of monomers through coil-coiled interactions. Two dimers assemble into a tetramer and eight tetramers form a unit length filament (ULF) which then assemble into a filament via an end-to-end annealing.

Supplemental Figure S1

Dabbaghizadeh et al.

A

MILKEVTSVVEQAWKLPESERKKIIRRLYLKWHPDKNPENHDIANE
VFKHLQNEINRLEKQAFLDQNADRASRRTFSTSASRFQSDKYSFQR
FYTSWNQEATSHKSERQQQNKEKCPPSAGQTYSQRFFVPPTFKSV
GNPLESRGPFEQKLISEEDLN

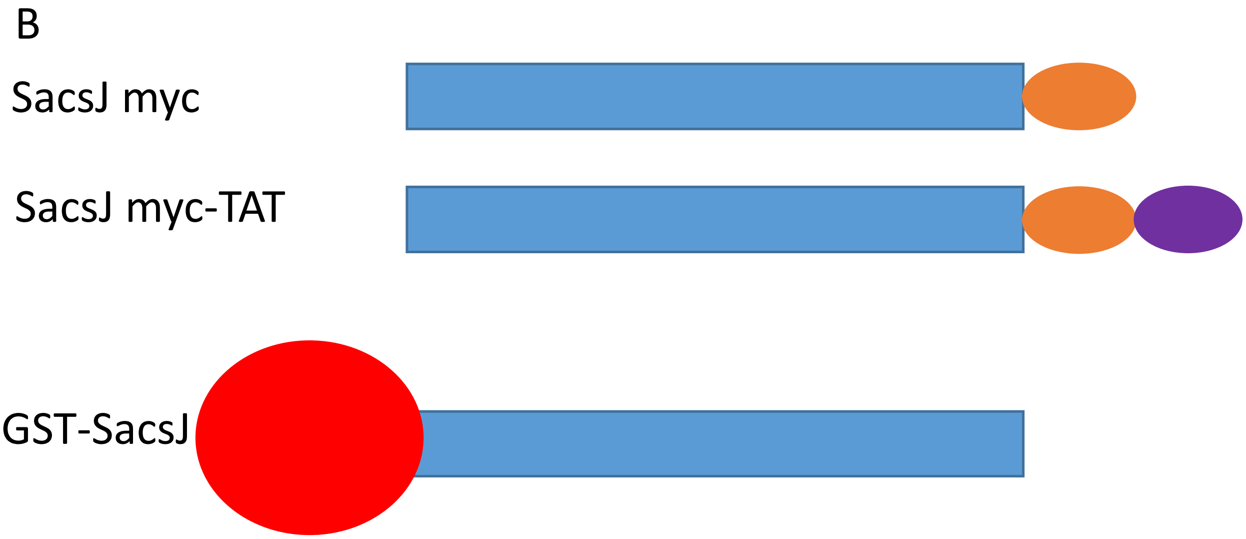

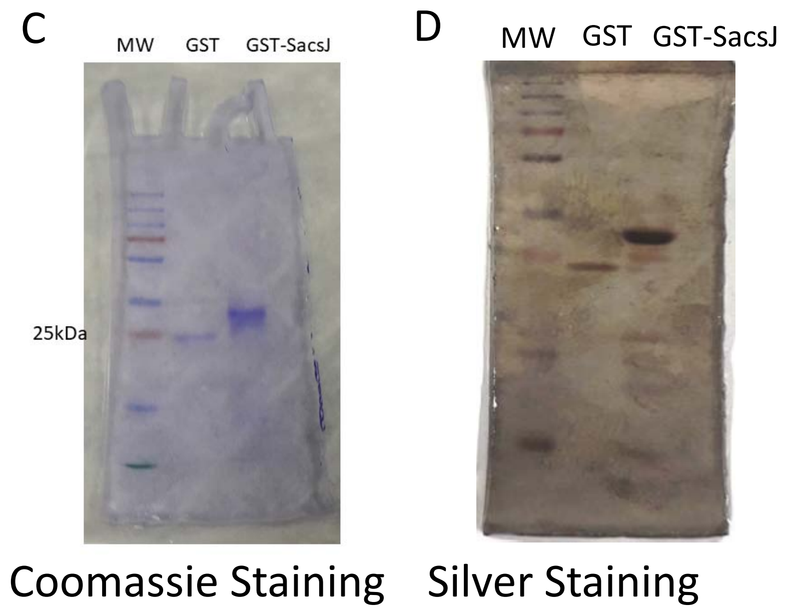

Fig S2 Supplemental. Sequence and diagram of SacsJ constructs used in this study. (A) Amino acid sequence of the SacsJ domain showing the conserved HPD sequence responsible of HSP70 binding, which was mutated to produce the SacsJ H33Q variant. (B) Diagram representing the different SacsJ constructs used: SacsJ domain fused in its C-terminal end to either myc or myc-TAT or N-terminally fused GST. (C and D) Commassie and silver staining of a SDS-PAGE analysis of 5 μg of GST and 25 μg of GST-SacsJ showing no major co-contaminants.

Supplemental Figure S2
Dabbaghizadeh et al.

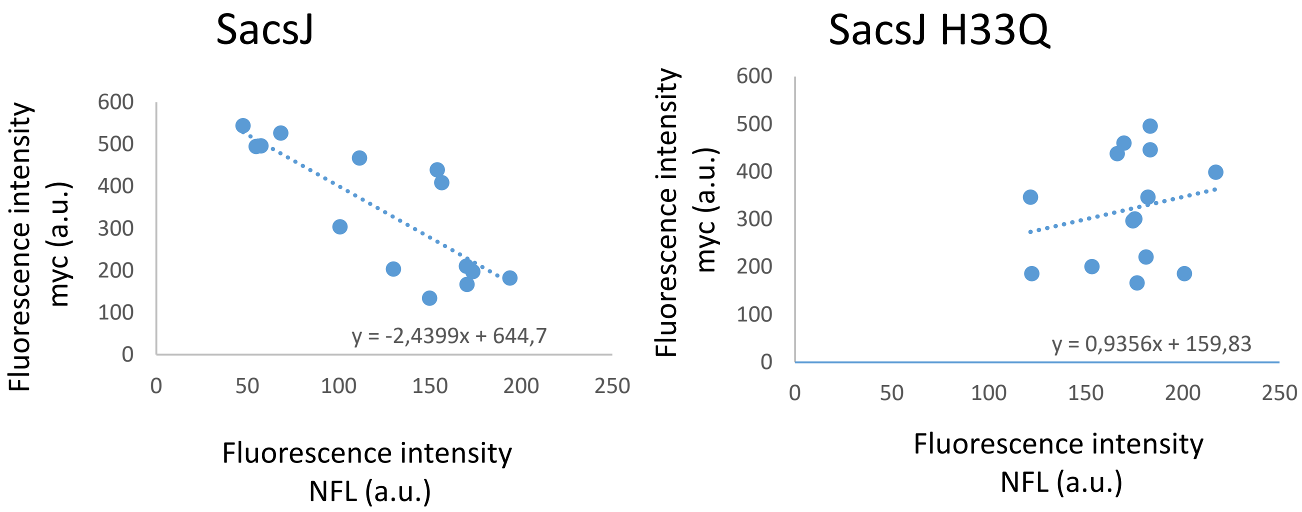

Fig S3 Supplemental. NFL degradation following expression of SacsJ-myc, but not SacsJH33Q-myc.

Graphical representation of fluorescence intensity (in arbitrary units) of SacsJ or SacsH33Q and NFL measured in *Sacs*$^{-/-}$ motor neurons in culture. Linear regression analysis showed a negative relationship between expression levels of SacsJ-myc with NFL, but no correlation with the expression levels of SacsJH33Q-myc. a.u.= arbitrary units, n=15.

Supplemental Figure S3
Dabbaghizadeh et al.
